# Quinine inhibits ovulation and produces oxidative stress in the ovary of cyclic Sprague-Dawley rats

**DOI:** 10.4314/ahs.v18i2.8

**Published:** 2018-06

**Authors:** Stella Chinwe Gbotolorun, Oghenevwakpeje Inikori, Olawande Damilola Bamisi, Abraham Adewale Adepoju Osinubi, Abayomi Olugbenga Okanlawon

**Affiliations:** Department of Anatomy, Faculty of Basic Medical Sciences, College of Medicine of the University of Lagos, Nigeria

**Keywords:** Quinine, ovary, LH, ovulation, oxidative stress

## Abstract

**Background:**

Quinine has been reported to possess anti-spermatogenic activities.

**Objectives:**

This study was carried out to determine the effect of quinine on ovarian function in Sprague-Dawley rats.

**Methods:**

Twenty rats with regular 4-days oestrous cycle divided into 4 groups (N=5) were used. Group I received quinine at 30 mg/kg body weight by gavage for 28 days after which they were sacrificed. The ovaries were excised for biochemical oxidation of glutathione peroxidase (GSH), superoxide dismutase (SOD), catalase and malondialdehyde (MDA). Group II received single dose quinine at 30 mg/kg body weight at 0900 hrs on day of proestrus. Blood was obtained at 1800 hrs for hormonal assay of FSH and LH. The animals were sacrificed the next morning on estrus: oviducts were examined for ova count. Groups III and IV served as controls.

**Results:**

Quinine treated rats recorded zero number of ova compared to control. Serum concentration of LH reduced significantly in the quinine treated group compared to the control. Furthermore, quinine significantly decreased the oxidant status of GSH, SOD and catalase and significantly increased MDA levels in the ovary compared to the control group.

**Conclusion:**

Quinine completely blocks ovulation, suppresses LH surge, and produces oxidative stress in the ovary.

## Introduction

Quinine is a cinchona alkaloid that belongs to the aryl amino alcohol group of drugs. Almost 400 years after Jesuit priests first documented its efficacy; quinine still remains a therapeutic anti-malarial drug as seen on the WHO Model List of Essential Medicines^1^. Quinine is still a potent drug in the treatment of the four species of malaria parasites, and is still the treatment of choice for cerebral malaria^2^ and nocturnal leg cramp^3^.

Increased production of reactive oxygen species (ROS) in the whole blood has been observed in patients with acute *falciparum* malaria^4^ and also in *P. vinckei* infected mice^5^. The administration of quinine to non-malarious individuals elicited a flux in erythrocyte lipid peroxidation; with an initial increase followed by a reduction in lipid peroxidation^6^. Quinine has been reported to generate ROS when photosensitized in the presence of UV-B radiation even within a cellular environment^7^.

Most of the in-vivo and in-vitro studies to determine the effect of quinine on the reproductive system and function have been carried out on males. The reports from some of these studies using animal models have shown that quinine possesses anti-spermatogenic activities: disrupts spermatogenesis, reduces sperm motility, morphology and sperm count and is deleterious to testicular histoarchitecture^8^–^10^.

There is a dearth of literature on the effect of quinine on the female reproductive system. This study was carried out to determine the effect of quinine on ovarian function in cyclic Sprague-Dawley rats.

## Materials and methods

### Animals

A total of thirty regular 4-day cycling female rats of Sprague-Dawley strain weighing between 120 – 200 g were used. The animals had access to food and water ad libitum. They were maintained at 25 ± 3°C with photoperiodicity of 12: 12 light: darkness. The animals were observed for clinical signs of drug toxicity throughout the duration of the experiment. All procedures involving animals in this study were approved by the Departmental Committee on the use and care of animals and tissue collection.

### Quinine

Quinine dihydrochloride injection, a product of Jiangsu, China, was given intramuscularly at a single dose of 30 mg/kg/day on the morning of proestrus to determine ovulation. To determine oxidative stress, quinine sulphate tablet a product of Wockhart was constituted into a solution by the addition of distilled water and administered at the same dose orally by gavage once daily for 28 days. Our choice of dosage selection was based on a previous study^8^. This dose falls within the no-effect level and is therefore grossly non-toxic^11^. Control animals received 1 ml of distilled water.

### Determination of ovulation

Ovulation studies were determined using the method of Kim et al^12^. Briefly, vaginal smear was taken daily to determine the stage of the oestrous cycle. Rats with a preponderance of uniformly nucleated cells indicating the proestrus phase, received a single dose of quinine intramuscularly at 9 a.m. Later the same day in the evening at 6 p.m, blood was collected via ocular puncture using a capillary tube and was stored in heparinized bottles. The next day, which was the day of estrus, the rats were sacrificed by cervical dislocation at 10 a.m. A ventral laparotomy was performed and the oviduct was excised, placed on glass slides with a drop of saline and covered with cover slips. It was squeezed with both sides being gently rocked. Any ovum found in the distended ampulla was counted under a light microscope.

### Hormonal assay

Blood was centrifuged and serum was decanted from the plasma and assayed in batches with control sera at both physiological and pathological levels with a microwell kit to determine FSH and LH concentrations.

### Oxidative stress study

Quinine was administered daily for 28 days by gavage^13^. At the end of the treatment period, the rats were sacrificed by cervical dislocation. A ventral laparotomy was performed and the ovaries were excised and kept frozen at −20°C until the day of assay for biochemical analysis of oxidative stress.

### Preparation of ovarian tissues for oxidative stress

The ovarian tissue was homogenized in a Teflon-glasshomogenizer with a buffer containing 1.5% potassium chloride to obtain 1:10 (w/v) whole homogenate. MDA was measured using the method described by Farombi et al^14^. The GSH level in ovarian tissue was estimated as described by Rukkumani et al^15^. Catalase was assayed colorimetrically at 620 nm and expressed as _mol of H_2_O_2_ consumed/min as described by Rukkumani et al^15^. Total SOD activity was measured by the method described by Sun et al^16^.

### Statistical analysis

Results were analyzed and expressed as mean ± standard error of mean (Mean ± SEM) and subjected to statistical analysis using paired sample t-test. Statistical significance was considered at *p< 0.05*.

## Results

### Effect on body weight and ovulation

Control rats gained weight in the course of the treatment and the gain in weight was significantly different at the end of the study. On the other hand the weight of the quinine treated rats reduced significantly by the end of the treatment (Table 1).

**Table 1:** Effect of administration of quinine on body weight and on ovulation in cyclic Sprague-Dawley rats

Treatment groups	Before (g)	After (g)	No. of ova shed in the oviduct
Control	162.4 ± 5.2	187 ± 4.6	7.6 ± 0.5
Quinine	185.2 ± 4.0	157.8 ± 6.8*	0.0 ± 0.0*

Values are expressed as mean ± S.E.M (N=5).

* *p*<0.05 vs. control.

In addition, quinine completely blocked ovulation as no ovum was observed in the oviduct in the morning of estrus. However, control animals recorded a count of 7.6 ± 0.5 numbers of ova.

### Effect on serum concentrations of FSH and LH

Serum concentrations of both FSH and LH (p<0.05) decreased when treated group was compared to the control values (Table 2).

**Table 2:** Effect of administration of quinine on serum levels of FSH and LH in cyclic Sprague-Dawley rats

Treatment groups	FSH (µlU/ml)	LH (µlU/ml)
Control	0.47 ± 0.07	1.17 ± 0.13
Quinine	0.38 ± 0.07	0.81 ± 0.05*

Values are expressed as mean ± S.E.M (N=5).

* *p*<0.05 vs. control.

### Effect on markers of oxidative stress

The study showed that MDA increased significantly while GSH, Catalase and SOD activities reduced significantly when compared to the control group (Table 3).

**Table 3:** Effect of administration of quinine on biochemical oxidation of MDA, GSH, catalase and SOD in cyclic Sprague-Dawley rats

TREATMENT GROUPS	MDA (µmole/ml/mg)	GSH (µmole/ml/mg)	CATALASE (µmole/ml/min/ mg pro)	SOD (µmole/ml/min/mg pro)
Control	2.63 ± 0.07	30.06 ± 0.40	12.78 ± 0.17	2.38 ± 0.35
Quinine	4.39 ± 0.04*	18.39 ± 0.53*	10.44 ± 0.53*	2.09 ± 0.08*

Values are expressed as mean ± S.E.M (N=5).

* *p*<0.05 vs. control.

## Discussion

Body weight decreased significantly in the quinine treated rats compared to the control rats in this study. This result is consistent with another study in which mice were fed quinine diet. The authors suggested that quinine diet caused weight loss in the mice as a result of de-regulation in intestinal nutrient absorption rather than energy expenditure^17^.

Numerous animal and human studies have demonstrated the presence of ROS in the female reproductive tract such as in the ovaries^18^–^20^, the fallopian tubes^21^ and in embryos^22^. ROS is involved in the modulation of an entire spectrum of physiological reproductive functions such as oocyte maturation, ovarian steroidogenesis, corpus luteal function and luteolysis^18^–^20^,^23^. In the reproductive tract, increased ROS generation is limited by a variety of anti-oxidant enzymes, such as SOD, catalase, and various peroxidases. A delicate balance exists between ROS and anti-oxidant enzymes in the ovaries. Anti-oxidant enzymes neutralize ROS production and protect the oocyte and embryo from apoptosis resulting from nucleic acid strand breaks, lipid peroxidation, protein degradation and ultimately, cell death^24^. SOD causes a dismutation of superoxide radicals to H_2_O_2_, which is further detoxified to water and oxygen by catalase or GSH.

Results from this study revealed that administration of quinine significantly elevated the level of MDA, and at the same time significantly reduced the levels of GSH, SOD and Catalase in the rat ovaries when compared to the control. The report of this study is in agreement with Osinubi et al.,^25^ who reported that quinine significantly increased levels of MDA in the testes. Also, in another study using amodiaquine, it was reported that amodiaquine caused a significant reduction in the enzymatic anti-oxidant activities of SOD and catalase in the ovaries of Sprague-Dawley rats^13^. An imbalance between ROS production and anti-oxidants results in oxidative stress. From the result of this study, it can be deduced therefore that the ovaries were under oxidative stress.

Certain drugs acting at various levels of the hypothalamic-pituitary-ovarian axis have been reported to impair ovulation and ultimately, fertility^26^. Ovulation is the accomplishment of a series of events orchestrated by the surge of LH. These events are characterized by resumption of meiosis, break down of germinal vesicle, granulosa luteinization, stigma formation and ultimately the release of a mature ovum^27^. In this study, the administration of quinine at 0900 hrs on the morning of proestrus blocked ovulation completely as not a single ovum was found in the oviduct compared to the control. The result of this study is in concert with other investigators who reported a complete block in ovulation with regression of LH surge when chloroquine was administered between 0900 and 1000 hrs on proestrus^28^,^29^. No significant difference was observed in the serum concentration of FSH however LH levels decreased significantly from control values. It is a well-established fact that on the afternoon of proestrus, about 2 to 3 p.m., the circulating levels of LH begins to increase rapidly and ultimately reach peak levels by 5 to 7 p.m. on that same evening. This proestrus surge of LH is responsible for the process of and events following ovulation. This surge of LH actuates follicular rupture and the resultant ovulation on the morning of estrus^30^. The significantly reduced levels of LH observed in this study revealed that the expected surge in LH that induces follicular rupture and eventual ovulation was suppressed.

As shown in our study above, quinine produces overproduction of ROS a fact suggested by the significantly increased levels of MDA an important lipid peroxidation marker. ROS represents the most important class of free radicals generated in living systems. ROS is generated in the form of hydrogen peroxide (H_2_O_2_), superoxide radical (O_2_•−), and hydroxyl radical (OH•). H_2_O_2_ is a weak oxidizing agent that can cross rapidly inside the cell membranes and damage intracellular systems^31^. Removal and neutralization of H_2_O_2_ by catalase and glutathione peroxidase is very important for the protection of living systems. Hydrogen peroxide is constantly produced in cells and has been implicated in several ovulation-related events such as steroid hormone production^32^,^33^. It has been shown that the addition of hydrogen peroxide to a culture of human chorionic gonadotropin-stimulated luteal cells resulted in reduction of both progesterone and estradiol production^20^. Estrogens and progesterone are important in the normal functioning of the female reproductive system. The balance in hormonal interplay between estrogens and progesterone is responsible for a normal regular cycle^34^,^35^. Several authors have demonstrated that progesterone secreted during the diestrus phase intensifies the negative feedback of estradiol on basal LH secretion and when the level of progesterone falls at luteal regression, estradiol and LH levels rise and ovulation occurs within the following 6–8 h^36^–^39^. Also, low levels of estrogen inhibit ovulation by delaying the estrogen triggering of LH surge.

Although, progesterone and estrogen levels were not determined in this study however, it may be inferred that the oxidative stress produced by the administration of quinine may have suppressed the production of progesterone and estrogen. The suppressed levels of progesterone and estrogen may have inhibited the threshold of the neural and/or hypophyseal mechanisms responsible for ovulatory LH release leading to a complete block in ovulation in the morning of estrus. Although the dose given in this study falls below the recommended therapeutic dose in humans^6^,^40^ however, it is too early to begin to extrapolate the findings of this study to humans. Further studies should be carried out to elucidate the exact mechanism of action by which quinine blocks ovulation and induces oxidative stress in rats. Howbeit, quinine should be taken with caution in women who are undergoing treatment for infertility.

## Conclusion

The study demonstrated that quinine suppressed LH surge, completely blocked ovulation and produced oxidative stress in Sprague-Dawley rats.

## Recommendation

We have attempted to demonstrate an oxidative stress pathway by which quinine blocks ovulation however; our study was limited because we failed to determine the levels of progesterone and estrogen. Further studies to determine progesterone and estrogen levels should be carried out in order to substantiate and or validate our findings.
